# MFI-Net: A multi-resolution fusion input network for retinal vessel segmentation

**DOI:** 10.1371/journal.pone.0253056

**Published:** 2021-07-12

**Authors:** Yun Jiang, Chao Wu, Ge Wang, Hui-Xia Yao, Wen-Huan Liu

**Affiliations:** 1 College of Computer Science and Engineering, Northwest Normal University, Lanzhou, China; 2 Columbia University in the City of New York, New York, New York, United States of America; Fuzhou University, CHINA

## Abstract

Segmentation of retinal vessels is important for doctors to diagnose some diseases. The segmentation accuracy of retinal vessels can be effectively improved by using deep learning methods. However, most of the existing methods are incomplete for shallow feature extraction, and some superficial features are lost, resulting in blurred vessel boundaries and inaccurate segmentation of capillaries in the segmentation results. At the same time, the “layer-by-layer” information fusion between encoder and decoder makes the feature information extracted from the shallow layer of the network cannot be smoothly transferred to the deep layer of the network, resulting in noise in the segmentation features. In this paper, we propose the MFI-Net (Multi-resolution fusion input network) network model to alleviate the above problem to a certain extent. The multi-resolution input module in MFI-Net avoids the loss of coarse-grained feature information in the shallow layer by extracting local and global feature information in different resolutions. We have reconsidered the information fusion method between the encoder and the decoder, and used the information aggregation method to alleviate the information isolation between the shallow and deep layers of the network. MFI-Net is verified on three datasets, DRIVE, CHASE_DB1 and STARE. The experimental results show that our network is at a high level in several metrics, with F1 higher than U-Net by 2.42%, 2.46% and 1.61%, higher than R2U-Net by 1.47%, 2.22% and 0.08%, respectively. Finally, this paper proves the robustness of MFI-Net through experiments and discussions on the stability and generalization ability of MFI-Net.

## 1 Introduction

The retina contains a large number of blood vessels and is the only vascular system in the body that can be viewed in depth using non-invasive means. Common diseases such as retinal arterial and venous occlusion, high blood pressure and diabetes will have symptoms on the retinal blood vessels, so that timely detection of changes in the length, width, curvature, branching pattern, and transparency of retinal vessels [1] would have a high chance of avoiding blindness due to these diseases [2]. Segmentation of retinal vessel images has become an important task in modern medically assisted treatment and diagnosis, and traditional segmentation means are not only labor-intensive and time-consuming but also have great variability in segmentation results and are difficult to achieve accurate segmentation [3]. With the development of computer hardware and software and the increasing maturity of related technologies, the use of deep learning methods for automatic segmentation of medical images has not only reduced the burden of related workers but also significantly improved the segmentation accuracy. In view of the increasing significance of deep learning for medical image segmentation, more and more professionals are studying and using deep learning methods for automatic segmentation of retinal vascular images.

Retinal vessels are difficult to segment from the background, pathological regions, and other noise due to illumination imbalance problems. Therefore, a large amount of work has been devoted to solving these problems while improving the segmentation accuracy of the network. The following major research results have been presented in recent years: Zhang et al. [4] proposed a matched filter segmentation algorithm to detect blood vessels by thresholding the response of the retinal image to the matched filter, which significantly reduced the false detection generated by the original matched filter. Wang et al. [5] transformed the retinal image using 2D Gabor wavelets of different scales and applied morphological reconstruction. Oliveira et al. [6] proposed an unsupervised segmentation method using Frangi filters and Gabor wavelet filters to enhance and segment retinal vessel images. Liskowski et al. [7] proposed a supervised segmentation algorithm, which performed contrast normalization and zero-phase whitening on retinal images with a strong ability to combat noise, but the segmentation accuracy was low and could not segment some of the capillaries. Aslani et al. [8] used a mixed feature vector to train a random forest classifier for a supervised retinal vessel segmentation task, and the accuracy on DRIVE and STARE reached 95.13% and 96.05%, but with low sensitivity. Marin et al. [9] proposed a novel supervised method for vessel detection in digital retinal images. This method uses neural networks for pixel classification and computes a 7-dimensional vector consisting of gray levels and moment invariance-based features for pixel representation, obtaining 94.52% accuracy on the DRIVE dataset. Dharmawan et al. [10] proposed a novel hybrid algorithm based on U-Net [11] for retinal vessel segmentation of fundus images. Alom et al. [12] designed a recursive convolutional neural network RU-Net based on U-Net and R2U-Net using recursive residual convolution. Zhang et al. [13] took full advantage of low-level features and high-level features to design a novel network and utilized infinite convolution to obtain multi-scale features. Li et al. [14] improved the U-Net network by using an attention mechanism that can improve the segmentation at capillaries and improve the segmentation accuracy at vessel boundaries.

At present, the research on the segmentation of retinal blood vessels has also made great progress, but the accuracy and performance of the segmentation results of most methods still have a lot of room for improvement. At the same time, the problems of blurred blood vessel boundaries, obvious noise and inaccurate segmentation of capillaries have not been effectively solved. In order to solve the problem of vascular edge blurring, we designed a novel multi-resolution and multi-scale fusion input module, aiming to enhance the ability of accurate segmentation of boundaries by extracting more and richer feature information in shallow layers. The fusion of encoder and decoder information in the network is also redesigned, and the fusion of shallow and deep features is reconsidered so that the segmentation map recovered from the upsampling process can introduce less noise and segment a more complete blood vessel.

In summary, we make the following four contributions:

1. We redesigned the skip connection part based on the U-Net network model so that the shallow and deep feature information is more fully aggregated to strengthen the network’s ability to resist noise. At the same time, we combine channel attention to strengthen the contribution of the main features to the network and suppress the influence of the diseased area on the network.
2. We designed a multi-resolution fusion input module to extract shallower coarse-grained feature information and enhance capillary segmentation, and use spatial attention and channel attention to enhance vessel boundaries to obtain a more accurate and clear retinal vessel segmentation map.
3. We conducted ablation experiments and comparative experiments on three datasets DRIVE, CHASEDB1 and STARE. Experimental results show that the segmentation performance of the network model proposed in this paper is better than the current popular network model.
4. We experimented and analyzed the stability and generalization ability of the network, and discussed the robustness of the network model. We also give the experimental data and visualization results of the cross-test to provide a reference for subsequent researchers.

## 2 Related work

With the proposal of various semantic segmentation network architectures, image segmentation, as a major research hotspot of computer vision, has also developed tremendously in recent years. Among the currently popular segmentation network models, networks with skip connection and Encoder-Decoder structures perform well in segmentation tasks, so a lot of work is carried out on the basis of this type of network model. Multi-scale input can improve the feature extraction ability of network models, so it is widely used in many research work. Next, this article will introduce these related work, mainly including the discussion of the encoder-decoder type segmentation network model and the multi-scale input network model. Finally, this article will explain the main content of our work and the difference from other existing work.

### 2.1 Segmentation network of encoder-decoder structure

As early as 1985, David et al. [15] made the first attempt on the auto-encoder algorithm of the encoder-decoder structure on the Boltzmann machine. In 2006, Hinton et al. [16] used and systematically introduced the auto-encoder in his research. The auto-encoder is composed of two parts: an encoder and a decoder. The encoder part extracts image features through down-sampling, and the decoder part gradually restores the image through up-sampling, and finally plays the role of image compression and image denoising. Because the auto-encoder has end-to-end characteristics, Long et al. [17] tried to merge the encoder-decoder structure into the convolutional neural network, and proposed the FCN network model, and applied it to the segmentation task. FCN is an early successful network model that uses convolutional networks for segmentation tasks and has been widely used in various semantic segmentation tasks. In the task of medical image segmentation, the dataset is often difficult to obtain, and the number of samples in the obtained dataset is usually small. Since medical images contain different types of physiological structures of the human body, most of the medical images are very complex, but the sample images in the same dataset are highly similar, so if observed in the entire sample space, the medical image sample has the characteristics of single structure and simple semantic information. Because of the limitations of the network structure and the particularity of medical images, FCN performs generally in most medical image segmentation tasks. In 2015, the U-Net network model proposed by Ronneberger et al. [11] solved this problem to a certain extent. U-Net deepens the network depth on the basis of FCN, uses more down-sampling and convolution operations in the encoder part to fully extract the feature information in the image, and uses four consecutive up-sampling in the decoder stage to restore The edges of the resulting image are finer. U-Net uses skip connections in the corresponding stages of the encoder and decoder to promote the transmission and fusion of semantic information, ensuring that more low-level feature information can be used when performing image restoration in the decoder part. Compared with the FCN network, U-Net can extract more feature information from a data set with a small number of samples, and accurately segment complex medical images. The encoder-decoder type segmentation network structure has been further developed. Because U-Net performs well in the segmentation task of medical images, a lot of related work has begun to use it as the basic network. Zhou et al. [18] proposed UNet++, which rethinks and designs the skip connection part of the U-Net network, and discusses the optimal network layer number of the U-Net network. The R2-UNet proposed by Alom et al. [12] combines U-Net with residual network and recurrent neural network and improves the network result by strengthening the extraction of low-level feature information. Oktay et al. [19] integrated the attention mechanism into U-Net, and improved the network by suppressing irrelevant areas in the picture and strengthening more useful features.

### 2.2 Multi-scale input network

The purpose of multi-scale fusion is to extract different features by processing feature maps of different scales. Low-resolution feature maps can extract more complete global feature information, and high-resolution feature maps contain more detailed information. Cai et al. [20] proposed a multi-scale convolutional neural network model MS-CNN for object detection, which performs different processing on features of multiple scales, and can obtain and use different feature information. The atrous spatial pyramid pooling (ASPP) proposed by Chen et al. [21] can capture objects and their context information at multiple scales. Huang et al. [22] designed a unique multi-scale network model to extract shallow and deep feature information, which is conducive to extracting more complete shallow feature information. Zhao et al. [23] extracted multi-scale feature information through pyramid pooling and used local and global context information to make pixel prediction more reliable. Because the structure of multi-scale fusion can extract richer features from the network model, the multi-scale input module is gradually applied to the segmentation network. Zhao et al. [24] used multi-scale input in their design of ICNet segmentation network model and achieved good results in the task of scene segmentation, proving that using multi-scale and multi-resolution input structure can improve the performance of the segmentation network model. Liu et al. [25] also used a multi-scale input structure in the optical coherence tomography (OCT) segmentation task, which effectively enhanced the segmentation ability. Jiang et al. [26] used multi-scale input in the network model to ensure the transmission of original image features and achieved good results in the task of retinal blood vessel segmentation.

### 2.3 Methods of this paper

Compared with the existing methods, this paper rethinks multi-scale input and skip connection, and creatively designs a multi-resolution fusion input (MR, Multi-Resolution) module and fully aggregated skip connection (FAS, Fully Aggregated Skip connection), proposed the MFI-Net segmentation network model. The multi-resolution fusion input structure can extract and transfer more complete superficial features, further optimize the boundaries of retinal vessel segmentation results, and alleviate the problem of missing capillaries segmentation. The highly aggregated jump connection part can aggregate and fully integrate multiple levels of semantic and feature information, which greatly reduces the noise in the segmentation result.

## 3 MFI-Net segmentation network

**Fig 1:**
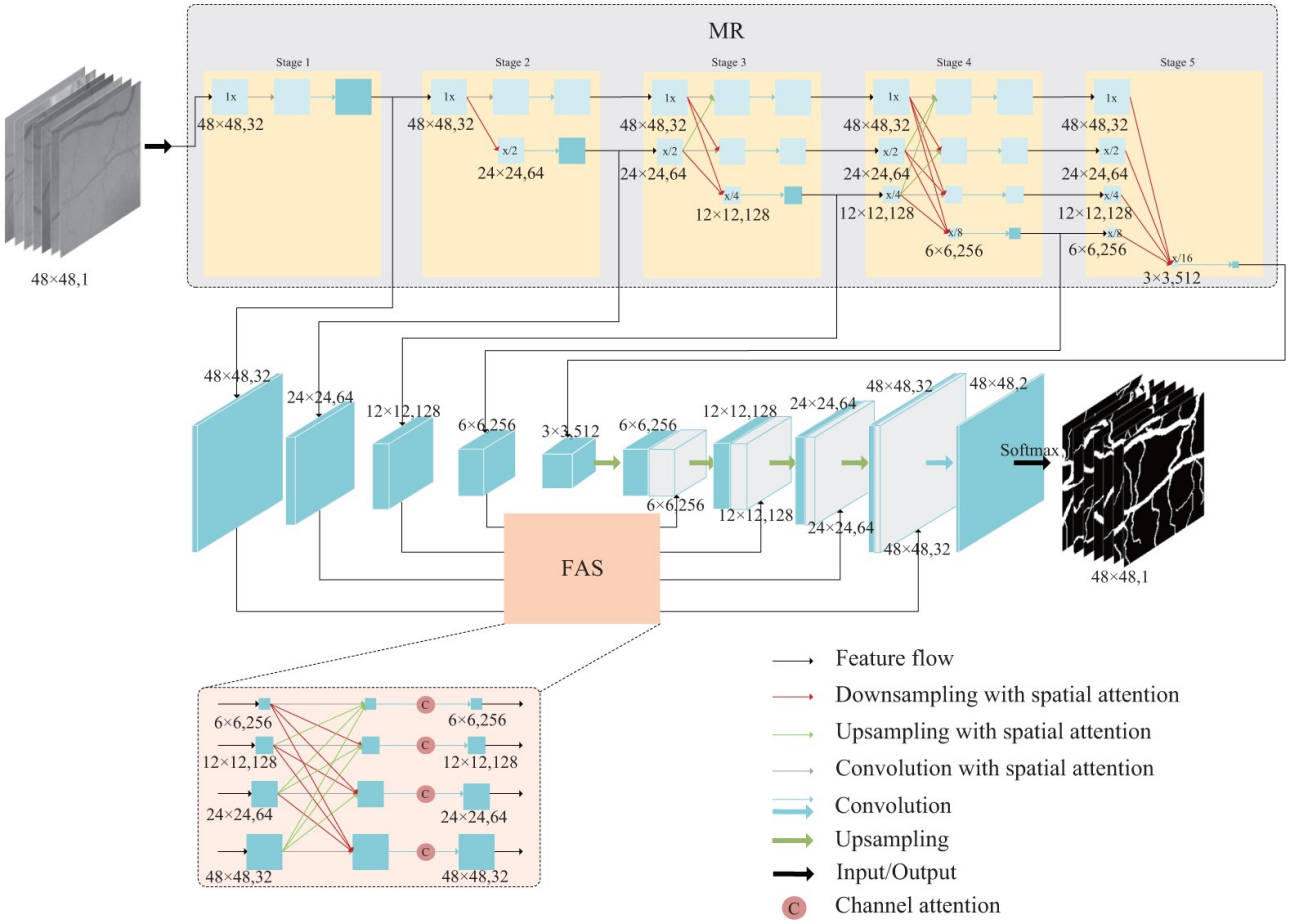
Overview of MFI-Net segmentation model for retinal vessel.

The MFI-Net proposed in this paper is shown in Fig 1. Input the 48×48 pixel retinal blood vessel medical image to the MR module, the MR module processes the original image, gradually fusion generates five resolution feature maps, and then obtains Five feature images with different resolutions are input to the encoder part of the segmentation network for further feature extraction. The decoder part gradually restores the segmentation graphics through upsampling, and each step of upsampling is spliced with the shallow encoder information processed by the FAS of the highly aggregated skip connection part. Finally, the segmentation result is obtained after 1×1 convolution and Softmax operation. Next, we will introduce the important parts of the model in detail.

### 3.1 Residual unit

**Fig 2:**
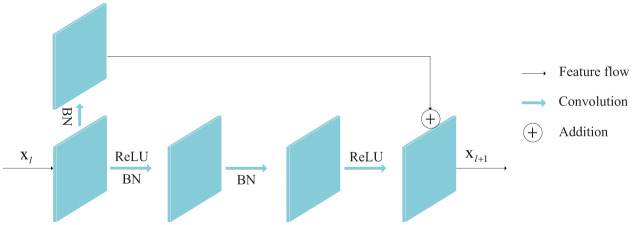
The structure of residual unit.

As the network structure continues to deepen, many networks will have the problem of gradient disappearance or gradient explosion, which leads to network degradation. In order to solve the above problems, He et al. [27] proposed a residual network. The residual unit is the basic structural unit of the residual network. Its structure is shown in Fig 2. The process can be expressed by Eqs (1) and (2).

$$\begin{array}{c} y_{l}=h(x_{l})+F(x_{l},W_{l}) \end{array}$$ (1)

$$\begin{array}{c} x_{l+1}=f(y_{l}) \end{array}$$ (2)

Where **x**_*l*_ and **x**_*l*+1_ are the input and output of the **l**-th residual unit, **l** represents the number of jumps, F is the residual function, which represents the learned residual relationship, and *h*(*x*_*l*_) = *x*_*l*_ represents the identity mapping, W_*l*_ represents weight, *f* is ReLU activation. Therefore, the features learned from shallow *l* to deep *L* can be represented by Eq (3).

$$\begin{array}{c} x_{L}=x_{l}+\sum_{i=l}^{L-1}F(x_{i},W_{i}) \end{array}$$ (3)

Inspired by He, this paper uses residual unit to replace ordinary convolution. Using the residual module to replace the ordinary convolution has the following advantages:

1. The residual network can be regarded as composed of multiple shallow networks, and there will be no network degradation inside each shallow network.
2. Because the residual module introduces a skip connection, the information in the network can flow into each residual unit without hindrance, which improves the information circulation capacity in the network.
3. In forward propagation, the feature information learned by the next layer must be equal to or more than the current layer.
4. In backpropagation, after introducing the residual module, each layer is more sensitive to changes in output, and the weight adjustment is more subtle.

### 3.2 Multi-resolution fusion input module

The multi-resolution fusion input module relies on the high-resolution image to gradually update the existing resolution feature image and generate the low-resolution feature image through repeated information exchange and information fusion in multiple stages. The process of updating the existing resolution feature image and the process of generating the new resolution can be represented by Eqs (4) to (6), where Eq (5) is the feature image updating process and Eq (6) is the new feature image generation process.

$$\begin{array}{c} A\left(x\right)=\{\begin{array}{c} 0,x=0\\ 1,x\neq 0 \end{array} \end{array}$$ (4)

$$\begin{array}{c} \begin{array}{cc} r_{m} & =A\left(m-1\right){\text{MaxPool}}^{2^{m-1},2^{m-1}}({\text{Conv}}^{3\times 3}(r_{1}))+\ldots \\ & +A\left(m-i\right){\text{MaxPool}}^{2^{m-i},2^{m-i}}({\text{Conv}}^{3\times 3}(r_{i}))+C(r_{m})\\ & +A\left(1\right){\text{TranConv}}^{2}(r_{m+1})+\ldots +A\left(i-m\right){\text{TranConv}}^{2^{i-m}}(r_{i}) \end{array} \end{array}$$ (5)

$$\begin{array}{c} \begin{array}{cc} r_{n+1} & ={\text{MaxPool}}^{2^{n},2^{n}}({\text{Conv}}^{3\times 3}(r_{1}))+{\text{MaxPool}}^{2^{n-1},2^{n-1}}({\text{Conv}}^{3\times 3}(r_{2}))+\ldots \\ & +{\text{MaxPool}}^{2^{n+1-i},2^{n+1-i}}({\text{Conv}}^{3\times 3}(r_{i}))+\ldots +{\text{MaxPool}}^{2,2}({\text{Conv}}^{3\times 3}(r_{n})) \end{array} \end{array}$$ (6)

Where *r*_*i*_ denotes the feature image of the *i*-th resolution, *r*_1_ denotes the image of the first resolution, i.e., the original input image. *r*_*m*_ denotes the current resolution, and *r*_*n*+1_ denotes the feature image of the new resolution. When *i* < *m*, the resolution of the feature image of the current operation is higher than the resolution of the target feature image that needs to be fused and updated, and the feature image is downsampled using a maximum pooling (MaxPool) with a stride of 2^*m*−1^ and a filter size of 2^*m*−1^ × 2^*m*−1^ and a convolution (Conv) of 3×3. When *i* = *m*, the resolution of the feature map of the current operation is the same as the resolution of the target feature map, and the feature map copy operation is performed. When *i* > *m*, the resolution of the feature map of the current operation is lower than the resolution of the target feature image, and the feature image of the current operation is upsampled using a transposed convolution (TranConv) with an upsampling rate of 2^*i*−*m*^.

In order to ensure that the features added in the feature map fusion update process do not have too much noise, this module uses a spatial attention mechanism [28] in the fusion stage to suppress areas of non-main features such as noise, focusing on the retinal blood vessel area. At the same time, channel attention [28] is used for the feature image after fusion to increase the contribution of the main feature to the network. Spatial attention and channel attention are defined by Eqs (7) and (8).

$$\begin{array}{c} M_{s}\left(r\right)=\sigma ({\text{Conv}}^{7\times 7}\left(\left[\text{AvgPool}\left(r\right);\text{Max}\;\text{Pool}\left(r\right)\right]\right)) \end{array}$$ (7)

$$\begin{array}{c} M_{c}\left(r\right)=\sigma \left(\text{MLP}\left(\text{AvgPool}\left(r\right)\right)+\text{MLP}\left(\text{MaxPool}\left(r\right)\right)\right) \end{array}$$ (8)

*r* represents the feature map, *σ* is the Sigmoid activation, and MLP is the multilayer perceptron. Therefore, the final mathematical expression of the update and generation of the feature map in the multi-resolution fusion input module is Eq (9), where ${\hat{r}}_{i}$ means that the feature map of the *i*-th resolution is processed into the current resolution feature map (the *m*-th resolution feature map Figure) Results after size.

$$\begin{array}{c} r_{m}={\text{M}}_{\text{c}}(\sum_{i=1}^{n}{\text{M}}_{\text{s}}({\hat{r}}_{i})) \end{array}$$ (9)

In simple terms, all feature maps above the current resolution are downsampled, all feature maps below the current resolution are upsampled, and the results are overlaid with the feature maps of the current resolution to update the current feature map. Therefore, the subsequent parallel feature map contains all the resolution feature information of the previous stage. In addition to updating the feature information of the existing resolution, a new feature map of lower resolution is generated, which contains all the important feature information of the existing resolution.

For a better understanding, we separately explain the second stage in the MR module in Fig 1. The previous stage generates two different resolution feature maps and passes them to the second stage, updates the 1x resolution feature map by stitching the x/2 resolution upsampled feature map and the 1x resolution feature map itself, updates the x/2 resolution feature map by stitching the x/2 resolution feature map itself and the 1x resolution feature map downsampled result and updates the x/2 resolution feature map by stitching the 1x resolution feature map downsampled result and the x/2 The new x/4 resolution feature map is generated by splicing the 1x resolution feature map downsampling result and the x/2 resolution feature map downsampling result, and then processed by the attention mechanism and input to the network or passed to the next stage, respectively.

### 3.3 Fully aggregated skip connections

As shown in the network structure in Fig 1, the skip-connected part FAS contains two parts: the information aggregation operation and the attention mechanism. The aggregation operation superimposes the feature maps of each resolution after up-sampling or down-sampling operations, similar to the information fusion part in the multi-resolution fusion input module, the aggregation approach in the FAS uses the information of the feature maps at different levels and then enhances the weight of the key information in the feature information after the channel attention. The output of each level of the FAS can be expressed by Eq (10), where *S*_*m*_ denotes the output information of the *m*-th level of the skip connection part.

$$\begin{array}{c} s_{m}={\text{M}}_{\text{c}}(\sum_{i=1}^{n}{\hat{r}}_{i}) \end{array}$$ (10)

## 4 Experiments and results

### 4.1 Dataset

**Fig 3:**
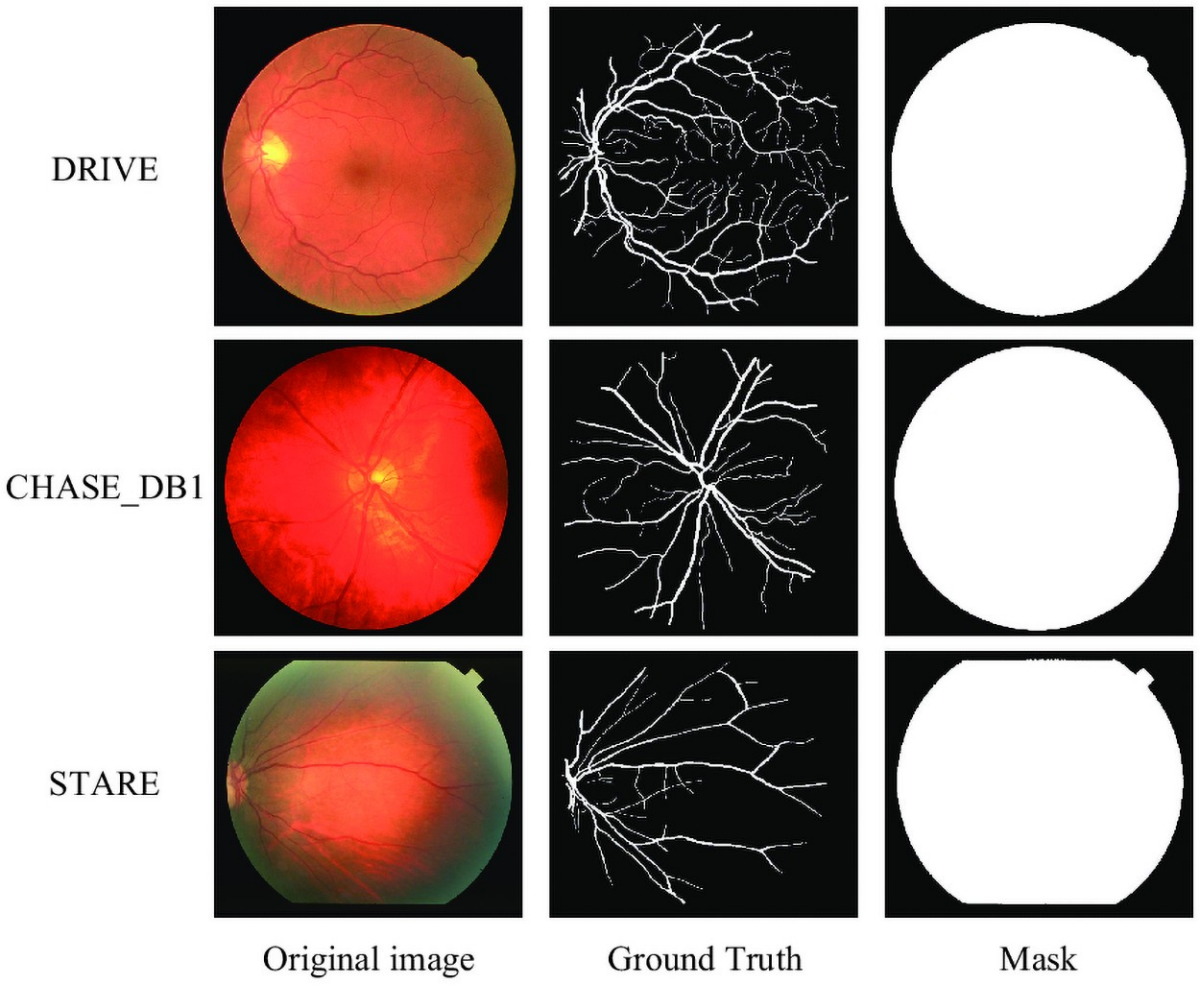
Sample images of DRIVE, CHASE DB1 and STARE datasets.

We conducted experiments on three mainstream retinal vascular image datasets, DRIVE [3], CHASE_DB1 [29] and STARE [30]. Fig 3 shows the sample images from the three datasets, including the original retinal vascular medical images, the resultant maps manually segmented by the physicians, and the masked maps.

The DRIVE dataset consists of 40 retinal images containing 7 pathology images. Twenty were taken as training samples and the other 20 as test samples. The size of the images was 565×584 pixels and these images were taken by a Canon camera at 45 degree field of view (FOV) [3]. Each image was manually segmented by two experts, and the manual segmentation result of the first expert was selected as the label.

The CHASE_DB1 dataset consists of 28 retinal images of 14 affected children. Twenty images were selected for training and another 8 images for testing. The size of the images was 960×999 pixels and these images were taken by the Nidek camera at a 30 degree field of view (FOV) [29]. Again, the segmentation result of the first of the two experts was chosen to be used as a label. Unlike the DRIVE images, the sample images in the CHASE_DB1 dataset have less visible blood vessels in the images due to uneven illumination.

The STARE dataset consists of 20 retinal images, including 10 pathological images. Due to the small number of samples in the data set, we use the leave-one-out method [31] for training and testing. The size of each picture is 700×605 pixels, and these pictures were taken by a TopCon camera in a 35 degree field of view (FOV) [30]. Similarly, the segmentation result of the first expert is selected as the label from the manual segmentation results of the two experts.

### 4.2 Pre-processing

**Fig 4:**
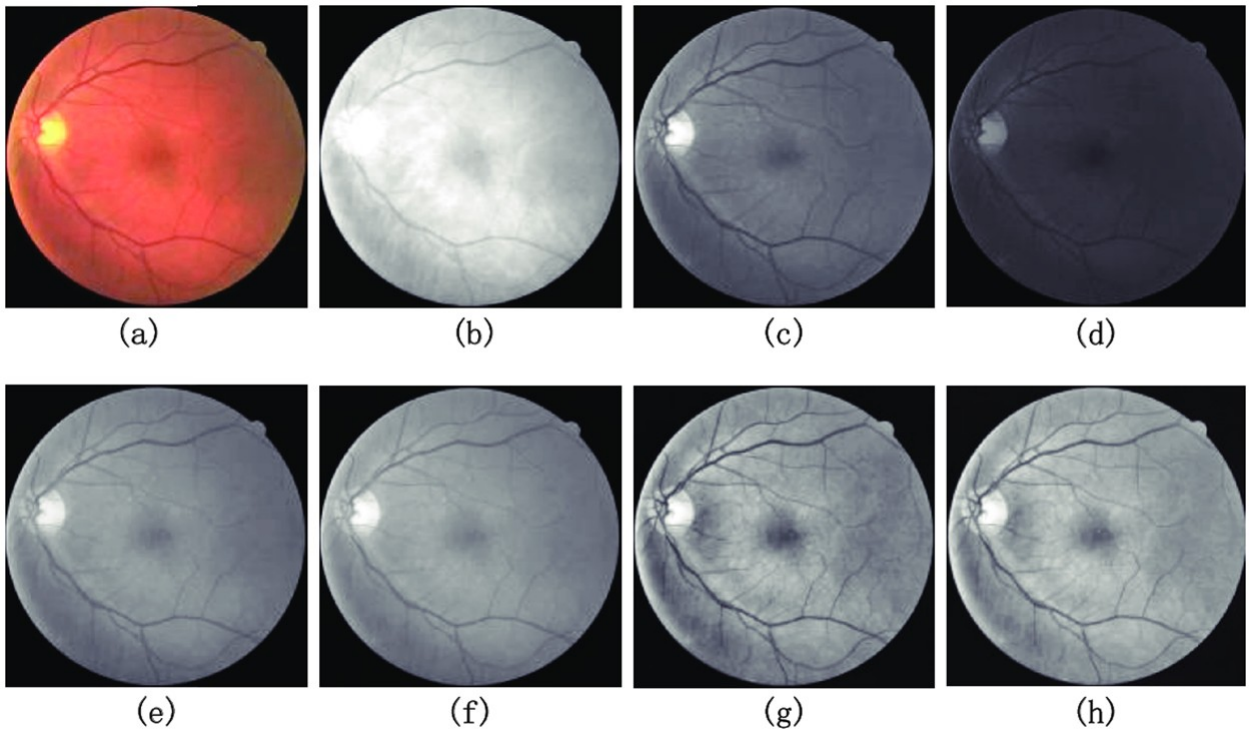
(a) is the original medical image. (b–d) are visualizations of red, green, and blue channels, respectively. Results of each preprocessing strategy. (e) is the image after grayscale processing. (f) is the image after data normalization. (g) is the image after CLAHE operation. (h) is the image after Gaussian correction.

In this paper, we adopted the preprocessing method proposed by Jiang et al. [26] for retinal images, and performed channel separation, grayscale processing, normalization processing, CLAHE processing, and gamma nonlinearization processing on the images. Fig 4 shows the results of the retinal images after the above mentioned treatments were applied to each of the retinal images, and it is clear that after the above preprocessing operations the contrast between the blood vessels and other parts of the retinal images is more obvious and the vascular parts become clearer, thus allowing our network to learn the data distribution of the images better when performing training [26].

It is necessary to perform channel separation before grayscale processing. Due to the individualization of fundus vascular images, the image noise of G-channel after channel separation is less and the vessels are clearer, which is more conducive to image segmentation. In Fig 4(a)–4(d) show the original image and the separated single-channel grayscale images of red, green and blue, respectively. It has been experimentally demonstrated [26] that the blood vessels are clearest in the grayscale images after the fusion of the red, green and blue channels according to the ratio of 29.9%, 58.7% and 11.4%. The results are shown in Fig 4(e). Fig 4(f) shows the image after grayscale processing of the graph, and there is a sharper contrast between the image area and the background. To further enhance the contrast, the contrast-constrained adaptive histogram equalization (CLAHE) method is used in the preprocessing to enhance the contrast between the effective regions and the background of the whole data set, as shown in Fig 4(g). Finally, gamma correction is used to further improve the image qual-ity, and the gamma value is set to 1.2, and the processing results are shown in Fig 4(h).

### 4.3 Evaluation indicators

To quantitatively evaluate the results of the proposed MFI-Net network in the fundus vascular segmentation task, we used several commonly used evaluation metrics to assess the overall performance of our method, including Sensitivity, Specificity, Accuracy, and F1. In the defined Eqs (11) to (16), TP denotes the number of pixels that label the vascular pixels correctly, TN denotes the number of pixels that label the background correctly, FP is the number of pixels that fail to label the vascular pixels correctly, and FN is the number of pixels that fail to label the background pixels correctly.

Sensitivity is defined by Eq (11), which indicates the percentage of pixels of correctly segmented vessels in the segmentation result map in the image. The sensitivity ultimately reflects the proportion of missed unsegmented blood vessel pixels in the segmentation result, and the closer the sensitivity is to 1.0, the less the missed unsegmented blood vessel fraction is and the better the segmentation effect is.

$$\begin{array}{c} Sensitivity=\frac{TP}{TP+FN} \end{array}$$ (11)

The definition of Specificity is shown in formula (12), which represents the proportion of mis-segmented blood vessel pixels in the image of the segmentation result image. Specificity ultimately reflects the size of the proportion of mis-segmented pixels. High specificity (close to 1.0) indicates that the fewer pixels that are incorrectly segmented in the segmentation result, the better the segmentation effect.

$$\begin{array}{c} Specificity=\frac{TN}{FP+TN} \end{array}$$ (12)

Accuracy (ACC) is defined as shown in Eq (13), which indicates the percentage of correctly segmented pixels in the segmentation result map (including vascular pixels and background pixels) in the whole segmentation map, reflecting the overall segmentation accuracy. However, since the pixel points in the black area of the segmentation result map account for the majority of the whole map, this index can reflect the segmentation result to a certain extent, but it cannot accurately evaluate the performance of the segmentation method.

$$\begin{array}{c} Accuracy=\frac{TP+TN}{TP+FP+TN+FN} \end{array}$$ (13)

F1 is a very common measure in binary classification models, which takes into account both Precision and Recall of classification models and is the summed average of Precision and Recall. Eqs (14) to (16) are the definitions of Precision, Recall and F1, respectively. When the F1 is higher (close to 1.0), it indicates better segmentation.

$$\begin{array}{c} Precision=\frac{TP}{TP+FP} \end{array}$$ (14)

$$\begin{array}{c} Recall=\frac{TP}{TP+FN} \end{array}$$ (15)

$$\begin{array}{c} F1=2\times \frac{Precision\times Recall}{Precision+Recall} \end{array}$$ (16)

### 4.4 Training

The CPU model of the machine used in the experiment is Intel(R) Xeon(R) Gold 5218 CPU@2.30GHz, the memory size at runtime is 187G, the GPU memory size is 24G, and the model is Quadro RTX 6000. The operating system used in the experiment is Linux, and the programming language used to build the network model is Python 3.7, the main library packages used are Pytorch 1.4, OpenCV 4.1.2, Numpy 1.18.1, and so on. All experiments are done in this experimental environment.

The setting of hyperparameters has a great influence on the performance and results of the experiment. The setting of hyperparameters in the experiment will be introduced below. For the DRIVE dataset with 40 images, 20 samples containing lesion images are selected as the training set, and the other 20 samples containing lesion images are reserved as the test set. Similar to the DRIVE data set, 20 images in the CHASE_DB1 dataset are used as the training set, and the other 8 images are used as the test set. Since there are only 20 images in the STARE dataset, in order to make the training effect as good as possible, the leave-one-out method was used for training in the experiment.

Dividing the dataset image into small patches is conducive to data expansion, can effectively reduce the risk of overfitting, and improve the performance of the model, so the dataset is divided into patches. Experimental verification shows that when the image is divided into patches of 48×48 pixels, the performance of the model can reach a better level. Both the training set and the test set are Divided. Due to the different sizes of images in different datasets, black pixels need to be used to complement the image to ensure that the image is accurately cut into integer patches.

For the DRIVE and CHASE_DB1 datasets, we set the training batch size to 128, the patch size to 48 pixels, the overlap sampling step to 5, the number of dynamically extracted patches to 10,000, and the threshold to 0.47. Since there are only 20 images in the STARE dataset, the leave-one-out method is used in the experiments in order to make the training effect as good as possible. The training batch size was set to 512, the patch size was set to 48 pixels, the overlap sampling step was set to 5, the number of dynamically extracted patches was set to 38,000, and the threshold was set to 0.52. The experiments on each dataset were conducted for 200 cycles, and the global random number of Pytorch was set to 1234. The default initialization method of the convolutional layer in Pytorch is used to assign the weight and bias in the network. The learning rate used in training was 0.001, and the Adam function was used as the optimizer of the network, where *β*1 = 0.9, *β*2 = 0.999, and *ε* = e-8 for Adam. In addition to using geometric transformation for data enhancement in the experiment, we also used the random local replacement algorithm used by Jiang et al. [26] in their work. To avoid overfitting while speeding up the training, we also used learning rate decay in our experiments [32, 33].

### 4.5 Experimental results

#### 4.5.1 Structural ablation

The MFI-Net proposed in this paper takes U-Net as the backbone network structure and creatively designs a new multi-scale and multi-resolution input structure to ensure that the coarse-grained feature information can be delivered and used to the maximum extent while improving the skip-connection part of the network to more fully fuse semantic information. We designed ablation experiments to verify the enhancement of MFI-Net by adding the MR module and the FAS module respectively to the U-Net network. In order to prove that the MR module has a boosting effect on the network, we also conducted experiments on the U-Net network with the addition of the traditional multi-scale input module MI (Multi-input Block).

Table 1 shows the experimental results of the structural ablation of each module, the bolded ones in the table is the best result. From the experimental results on the three datasets, the U-Net network with the addition of the MR module shows a significant improvement in all metrics compared to the U-Net network with the addition of the MI module, with an improvement of 1.04% in the F1 on the DRIVE dataset, and a significant improvement in the F1 on both the STARE and CHASE_DB1 datasets. Analysis of the experimental results on the DRIVE, CHASE_DB1 and STARE datasets showed that the FAS module had a huge improvement on the U-Net network, with F1 increasing by 1.60%, 2.42% and 1.54% on the three datasets, respectively.

**Table 1:** Ablation experiment results.

	DRIVE				CHASE_DB1				STARE			
Model	F1	Acc	Sen	Spe	F1	Acc	Sen	Spe	F1	Acc	Sen	Spe
UNet	0.8076	0.9660	0.8138	0.9806	0.7904	0.9740	0.7774	**0.9872**	0.8302	0.9743	0.8424	0.9852
UNet+MI	0.8130	0.9696	0.7544	**0.9902**	0.8083	0.9756	0.8134	0.9865	0.8382	0.9748	0.8709	0.9832
UNet+FAS	0.8236	0.9702	0.7950	0.9870	0.8146	0.9761	0.8319	0.9858	0.8456	0.9765	0.8632	0.9857
UNet+MR	0.8234	0.9704	0.7833	0.9885	0.8129	0.9758	**0.8347**	0.9853	0.8413	0.9752	**0.8797**	0.9829
UNet+MR+FAS	**0.8318**	**0.9705**	**0.8325**	0.9838	**0.8150**	**0.9762**	0.8309	0.9860	**0.8483**	**0.9766**	0.8619	**0.9859**

By comparing the enhancement effects of the MI and MR on the U-Net network in Table 1, it can be seen that the MR module has a greater improvement in the segmentation ability of the underlying model. MFI-Net (using the MR module and FAS module jointly) is at a higher level of sensitivity and specificity metrics compared to other ablation structures, with the best F1 and accuracy rates. The MFI-Net showed 2.42%, 2.46% and 1.81% improvement in F1 on the DRIVE, CHASE_DB1 and STARE datasets, respectively, and 0.45%, 0.22% and 0.23% improvement in ACC, respectively, compared with the benchmark network U-Net, further demonstrating the effectiveness of our proposed modules for network enhancement.

**Fig 5:**
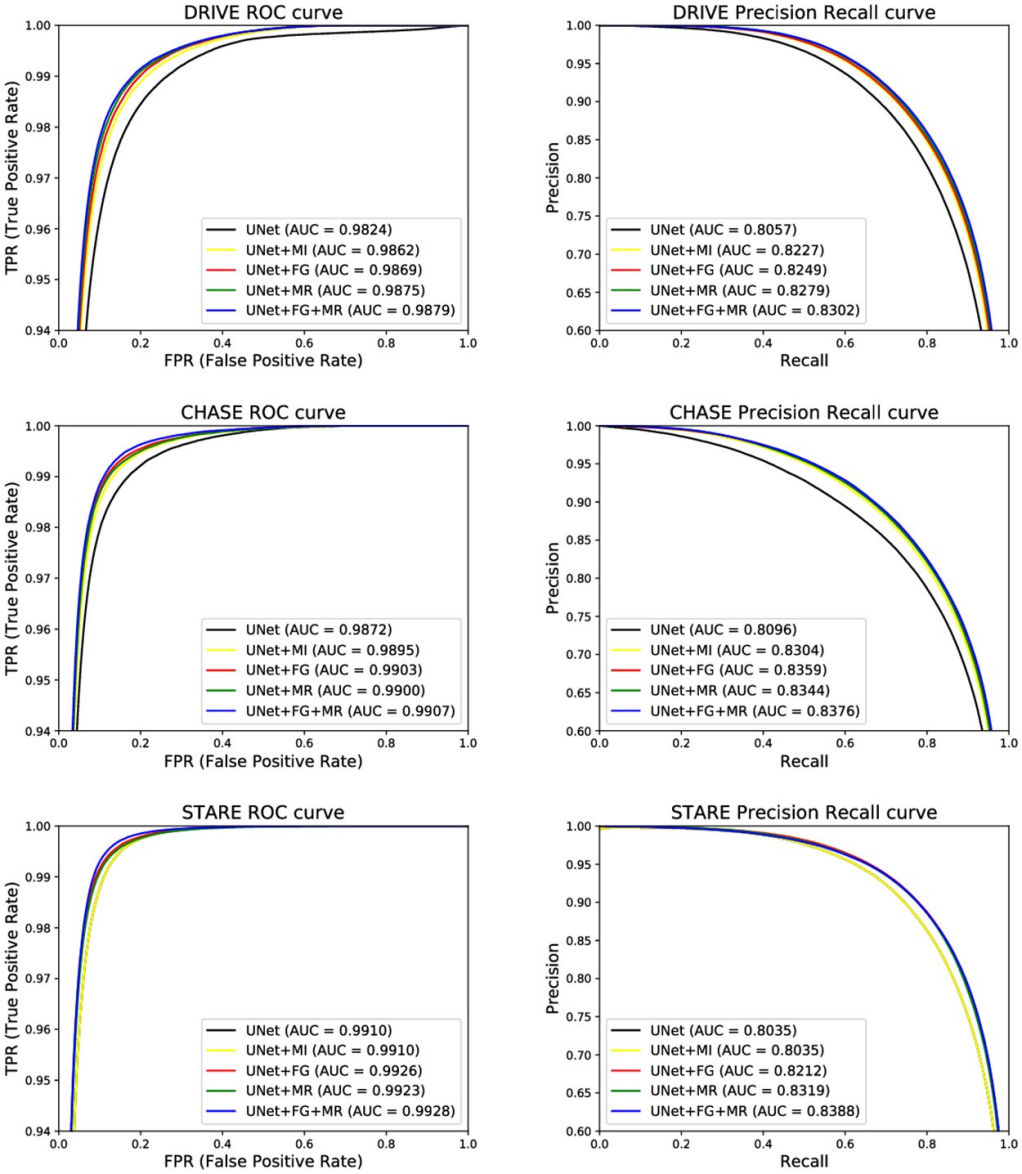
PR and ROC curves of each ablation structure.

Fig 5 calculates and visualizes the ROC curve and PR curve for each network model of structural ablation. The ROC curve reflects the information between the false positive examples (background pixels incorrectly segmented as vascular pixels) and the true positive examples (vascular pixels correctly segmented). The PR curve better reflects the true performance of the classification when the ratio of true positive examples to false positive examples is larger. The results show that the ROC values and PR values obtained from the U-Net network using both FAS and MR modules are the largest on both DRIVE dataset and STARE dataset and CHASE_DB1 dataset, indicating that this model obtains the best performance to better convey shallow feature information while extracting more deep feature information and better segmenting the blood vessels.

To demonstrate that the results obtained from our ablation experiments are statistically significant, we did a P-value analysis of the F1 metric in the segmentation results of each network model in the ablation experiments. We proposed the hypothesis that the former of the two given models has a better F1 than the latter on the given dataset. The results of the P-value analysis for this hypothesis are shown in Table 2.

**Table 2:** P-value analysis results among various ablation models on different dataset.

	**UNet:UNet+MI**	**UNet:UNet+FAS**	**UNet:UNet+MR**
DRIVE	0.0042	0.0035	0.0028
CHASE_DB1	0.0049	0.0093	0.0109
STARE	0.0146	0.0072	0.0115
	**UNet:UNet+FAS+MR**	**UNet+FAS:UNet+FAS+MR**	**UNet+MR:UNet+FAS+MR**
DRIVE	0.0043	0.0014	0.0273
CHASE_DB1	0.0081	0.0088	0.0053
STARE	0.0065	0.0219	0.0177

In Table 2, the probability of occurrence of the sample observations or more extreme results obtained when the original hypothesis is true for each ablation structure after adding different modules is less than the significance level 0.05. According to statistical principles, we are justified to reject the original hypothesis and believe that the F1 of the former is better than the latter for the network structures under comparison, i.e., the enhancement of the network by adding MI, FAS, and MR modules to U-Net is effective, and the results of using FAS module and MR module at the same time are optimal.

**Fig 6:**
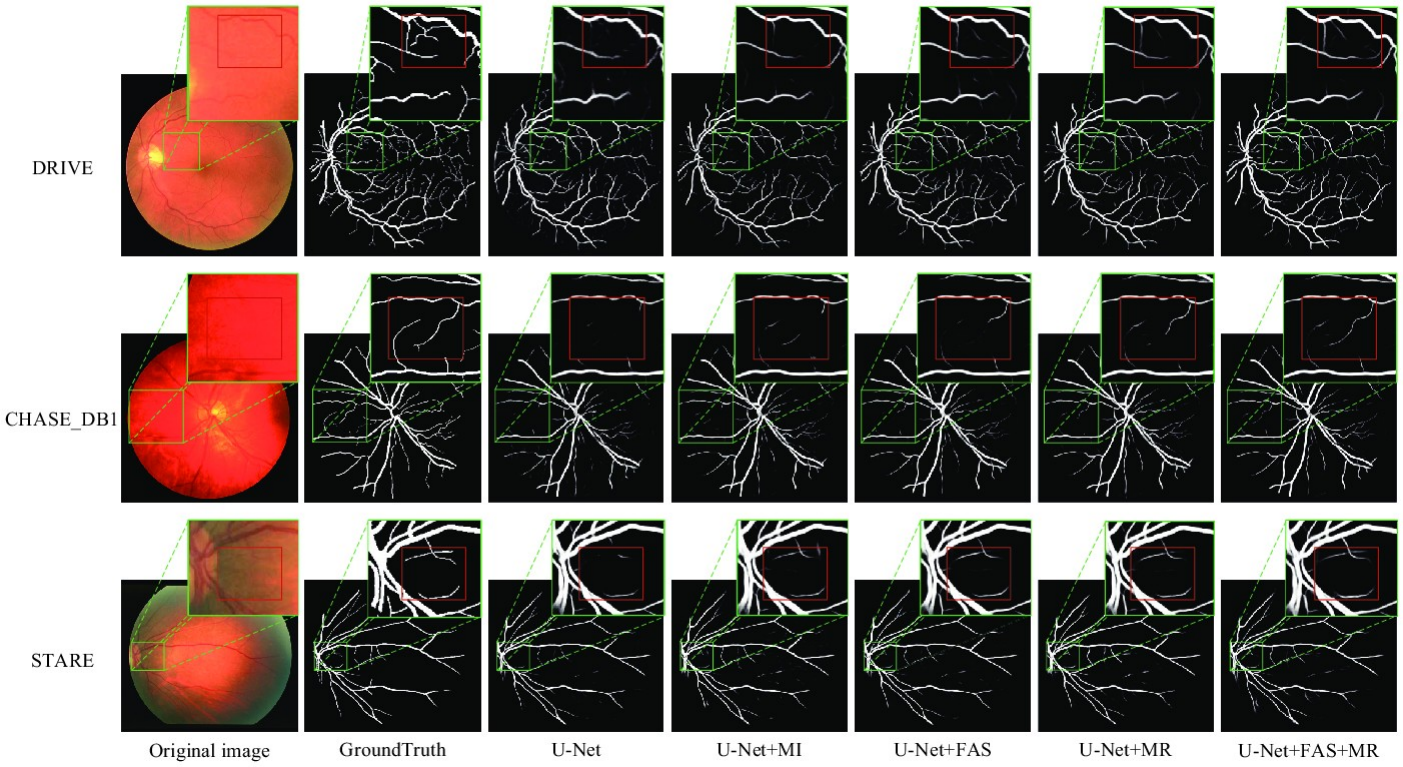
Segmentation result of ablation experiment. The areas that require special attention are zoomed in, and areas with obvious differences are marked with red rectangles.

The visualized images can show our segmentation results more intuitively, so in this paper, we visualize and compare the segmentation results of each ablation structure on each of the three datasets. The segmentation results of U-Net, U-Net with MI module added, U-Net with FAS module added, U-Net with MR module added, and U-Net with both FAS and MR modules are shown in Fig 6, respectively. For the segmentation task of fundus vessels, arterioles (the thicker vessels in the figure) are easier to segment, while the segmentation of capillaries is often not accurate enough. Therefore, we focused on the capillaries in the segmentation results for comparison. The first column of the figure shows the original retinal medical image, the second column shows the manually segmented labeled map, and the third to seventh columns show the segmentation results of U-Net, U-Net with MI module added, U-Net with FAS module added, U-Net with MR module added, and U-Net with both FAS module and MR module added (i.e., MFI-Net), respectively. From top to bottom are the medical image maps or segmentation result maps on the DRIVE, CHASE_DB1 and STARE datasets, respectively. For easy comparison, we have enlarged some areas in the retinal images, labeled images and segmentation result maps, and the focused observation areas are marked using red boxes.

As can be seen in Fig 6, for the thicker arterial vessels, the above network models are able to segment them clearly, but at the capillaries, there are certain differences in the segmentation results. Compared with the backbone network U-Net, adding the common multi-input module MI does improve the segmentation results of the network to a certain extent but compared with the segmentation results obtained from the network with the MR module, there are still many capillaries that are not segmented, which proves that the multiple resolution input images provided by MR can provide more feature information to the network, enhance the accuracy of the boundary, and improve the accuracy of capillary segmentation It has positive significance for the improvement of segmentation accuracy. Comparing the segmentation results of the base network U-Net and the U-Net using the FAS module, it is obvious that the latter has higher segmentation accuracy at fine details and less noise in the segmentation results, which proves that the information fusion of the FAS module is effective. Overall, during the process of structural ablation, the accuracy of the segmentation results gradually improved, the noise was significantly reduced, and both the boundary part and capillary part of the vessels were segmented more precisely and clearly, and the contrast of the areas marked in the figure was especially obvious. The above facts show that the network structure proposed in this paper is feasible and effective in a real segmentation task, and the improved network model can obtain better segmentation results.

#### 4.5.2 Model comparison

The experiment is also designed to compare the segmentation results of MFI-Net and the current popular fundus blood vessel segmentation network models such as UNet++, M2-UNet and AA-UNet on the three data sets of DRIVE, CHASE_DB1 and STARE. Tables 3 to 5 are the segmentation results of different model methods on three data sets. We have summarized the four evaluation indicators of F1, accuracy, sensitivity, and specificity. It can be seen from the table that compared with other model methods, MFI-Net has relatively good performance in various indicators. On the DRIVE dataset, our method can increase F1 by 0.16% to 1.47%, with a high level of accuracy, sensitivity and specificity. On the CHASE_DB1 dataset, our method can increase F1 by 0.11% to 2.67%, the accuracy rate can be increased by 1.54%, and the sensitivity can be increased by 5.53%. The F1 and accuracy of our method on the STARE data set have been improved, the F1 has increased by at least 0.08%, and the accuracy has increased by at least 0.13%.

**Table 3:** Segmentation results of different models on the DRIVE dataset.

Model	Year	F1	Accuracy	Sensitivity	Specificity
UNet++ [18]	2018	0.8302	**0.9710**	0.8120	0.9861
R2U-Net [12]	2018	0.8171	0.9556	0.7792	0.9813
CSNet [34]	2019	-	0.9632	0.8170	0.9854
Vessel-Net [35]	2019	-	0.9578	0.8038	0.9802
D-Net [36]	2019	0.8246	0.9709	0.7839	**0.9890**
D-UNet [37]	2019	0.8237	0.9566	-	-
M2UNet [38]	2019	-	0.9630	-	-
CTF-Net [39]	2020	0.8241	0.9567	0.7849	0.9813
AA-UNet [40]	2020	0.8216	0.9558	-	-
RCED-Net [41]	2021	-	0.9649	0.8252	0.9787
MRA-UNet [42]	2021	0.8293	0.9698	**0.8353**	0.9828
MFINet(Ours)	2021	**0.8318**	0.9705	0.8325	0.9838

**Table 4:** Segmentation results of different models on the CHASE_DB1 dataset.

Model	Year	F1	Accuracy	Sensitivity	Specificity
UNet++ [18]	2018	0.8139	0.9760	0.8184	0.9810
R2U-Net [12]	2018	0.7928	0.9634	0.7756	0.9820
Vessel-Net [35]	2019	-	0.9661	0.8132	0.9814
D-Net [36]	2019	0.8062	0.9721	0.7839	**0.9894**
D-UNet [37]	2019	0.7883	0.9610	-	-
M2UNet [38]	2019	-	0.9703	-	-
AA-UNet [40]	2020	0.7892	0.9608	-	-
RCED-Net [41]	2021	-	**0.9772**	**0.8440**	0.9810
MRA-UNet [42]	2021	0.8127	0.9758	0.8324	0.9854
MFI-Net(Ours)	2021	**0.8150**	0.9762	0.8309	0.9860

**Table 5:** Segmentation results of different models on the STARE dataset.

Model	Year	F1	Accuracy	Sensitivity	Specificity
UNet++ [18]	2018	0.8393	0.9753	**0.8646**	0.9843
R2U-Net [12]	2018	0.8475	0.9712	0.8298	0.9862
CSNet [34]	2019	-	0.9752	0.8816	0.9840
D-UNet [37]	2019	0.8143	0.9641	-	-
AA-UNet [40]	2020	0.8142	0.9640	-	-
RCED-Net [41]	2021	-	0.9659	0.8397	0.9792
MRA-UNet [42]	2021	0.8422	0.9763	0.8422	**0.9873**
MFI-Net(Ours)	2021	**0.8483**	**0.9766**	0.8619	0.9859

**Fig 7:**
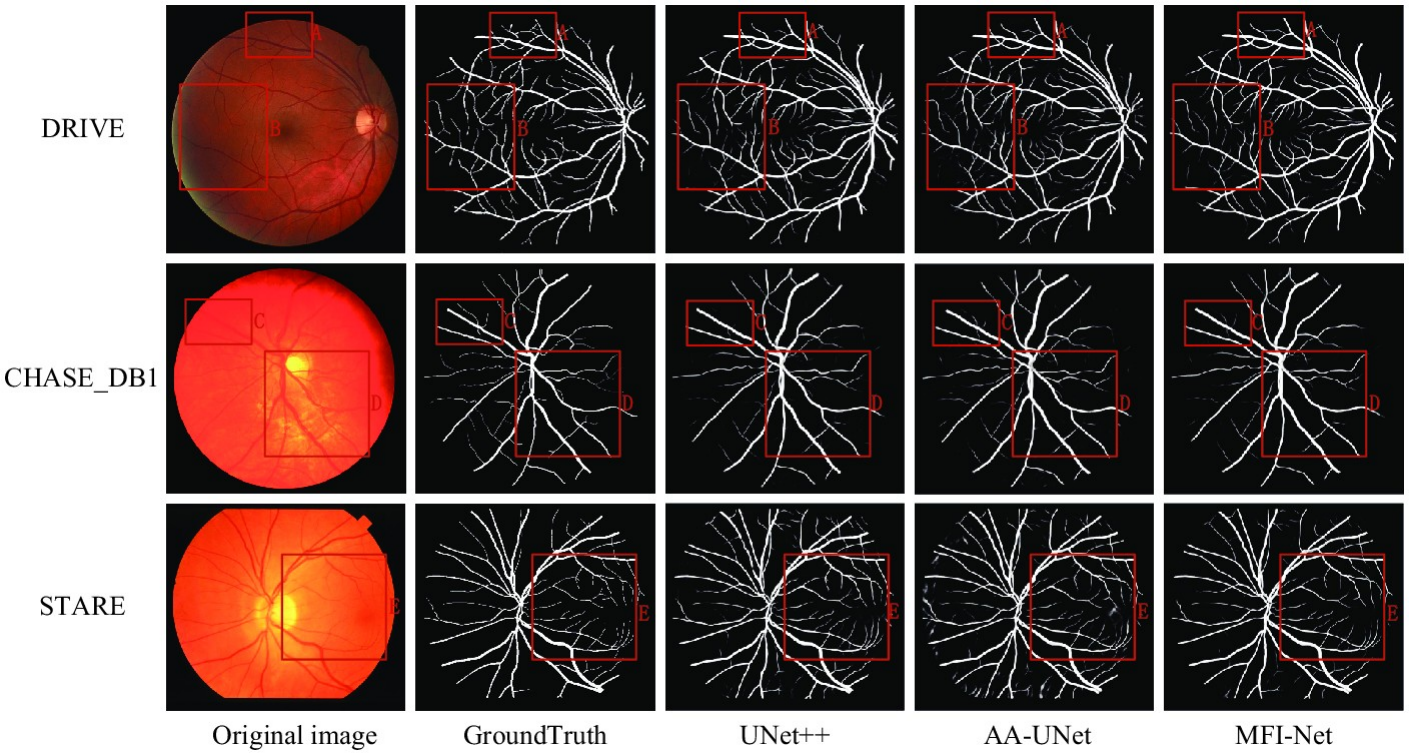
Comparison of segmentation results of MFI-Net(ours), UNet++ and AA-UNet. The red rectangles mark areas with obvious differences.

In order to compare and evaluate the MFI-Net performance more visually, we implemented the UNet++ network model and AA-UNet network model, which are still very popular, and applied them to the segmentation task of retinal vessels. Fig 7 shows the visualization of UNet++, AA-UNet and MFI-Net segmentation results.

On the DRIVE dataset and CHASE_DB1 dataset, some blood vessels are not segmented in the segmentation result map of UNet++, and there is obvious noise present in the segmented image of AA-UNet, and the segmentation is relatively blurred at the capillaries. Since MFI-Net has fully fused the feature information between the encoder and decoder, and utilized multi-resolution input to reduce the loss of coarse-grained information at the lower layers of the network when extracting features, it can extract more comprehensive features and segment more complete blood vessels. The low sensitivity of UNet++ and the relatively high sensitivity and specificity of MFI-Net are also shown correspondingly in Table 3, the bold ones of the table is the best result. UNet++ uses a large number of convolution and upsampling operations in the skip connection part, which greatly deepens the perceptual field of the network and enables the network to extract more feature information so that relatively clearer segmentation results can be obtained in the special region of the lesion, and this The segmented images of AA-UNet appear to be inaccurately segmented and noisy. In contrast, our method uses fewer computational resources compared to UNet++ but achieves better segmentation results.

Combining Tables 3 to 5 and Fig 7, we can conclude that the MFI-Net model performs well in all evaluation indexes. while focusing on the extraction of shallow features, MFI-Net focuses on amplifying the influence of important information, which not only effectively alleviates the loss of information of shallow features, but also focuses on the key features. And we use a more reasonable information fusion method to pass the useful information from the encoder to the decoder layers and minimize the noise in the segmentation results. Through the analysis of the segmentation results, our proposed method has better performance and is more advantageous in practical segmentation applications.

#### 4.5.3 Model parameter quantity and computation time analysis

To evaluate the spatial and temporal spending of our network models, we calculated the number of parameters for each ablation network structure as well as UNet++ and AA-UNet and recorded the training time of the above network models on different datasets and the segmentation time of the models on a single image in the same experimental setting. The details of the number of parameters and time overhead for each model are shown in Table 6. According to the data in Table 6, we can see that UNet is at a low level of time overhead and number of parameters due to its simple structure. On the contrary, UNet++, AA-UNet and MFI-Net use more modules and a more dense structure, which makes the number of network parameters relatively large and the time overhead is also higher. Combining Tables 3 to 5, we conclude that although UNet++ has relatively higher segmentation results compared to other network models, the large number of parameters and more time overhead often make it difficult to accept. The number of parameters and time spent are smaller and the results are better than those of UNet++ and AA-UNet.

**Table 6:** Details of the parameter amount and time cost of different models.

	DRIVE(10000 Pathcs)		CHASE_DB1(10000 Pathcs)		STARE(38000 Patchs)	
MODEL(Params)	training	test	training	test	training	test
UNet(7.8M)	5.57	1.68	5.52	5.43	20.32	5.28
UNet+MI(9M)	6.49	1.96	6.49	6.26	24.07	5.87
UNet+FAS(17.6M)	11.65	3.79	11.46	12.23	43.86	8.01
UNet+MR(18.2M)	13.82	3.92	14.07	12.63	52.54	10.01
UNet++(36.1M)	21.75	7.97	21.85	24.57	83.07	18.24
AA-UNet(28.3M)	19.97	6.21	20.21	21.01	78.84	15.89
MFI-Net(27.6M)	19.68	5.96	19.94	18.28	74.31	13.25

### 4.6 Robustness testing

#### 4.6.1 Testing on a single image

**Fig 8:**
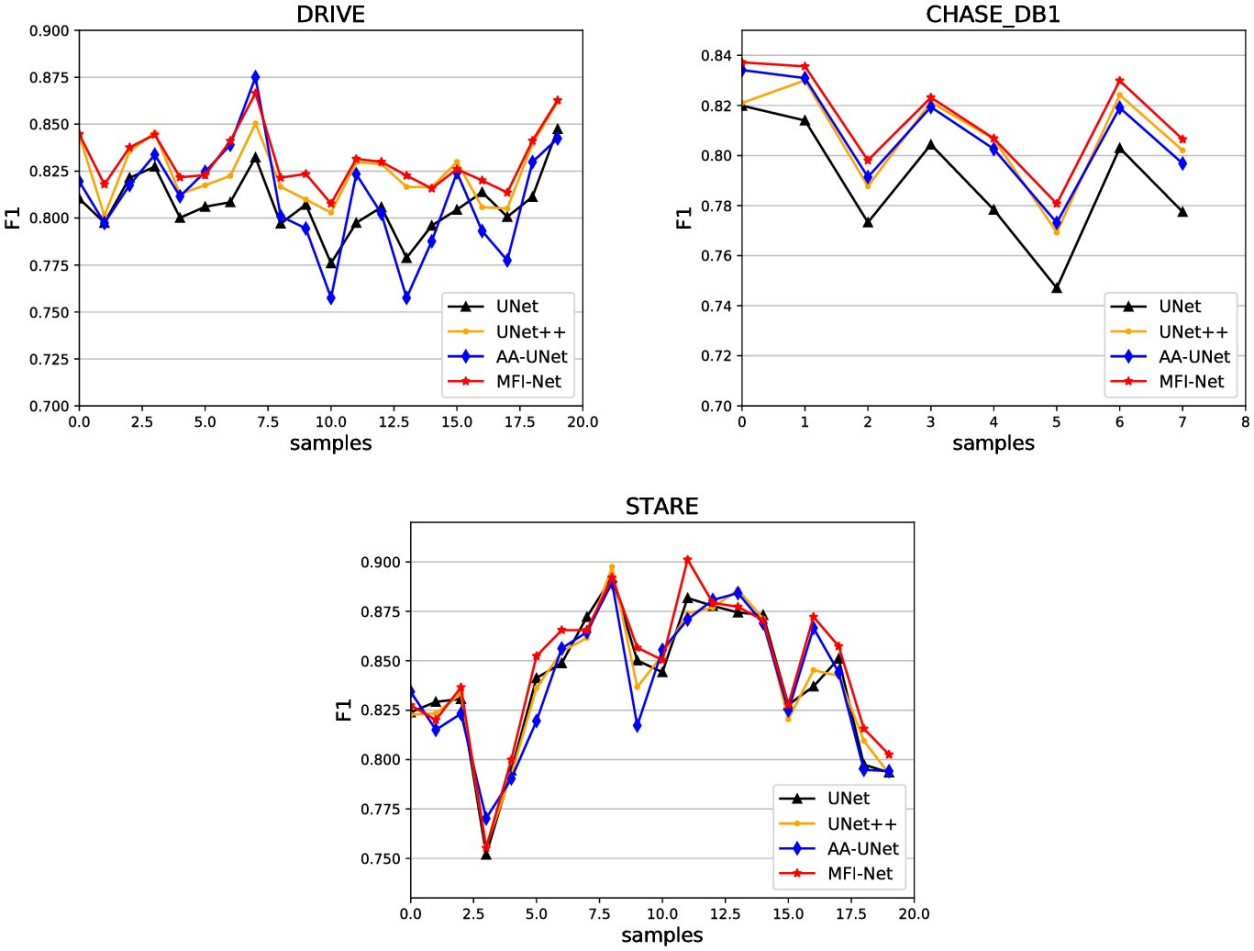
F1 performance curve on three datasets. The abscissa represents the sample picture number in the dataset, the ordinate represents the F1 for semantic segmentation of the image.

Fig 8 shows the F1 performance evaluation curves of MFI-Net and U-Net, UNet++ and AA-UNet for each image in the three datasets to further observe the performance of the network in the task of a single image. As can be seen from the figure, on the three datasets, the method in this paper is able to extract feature information stably, and perform to maintain a relatively stable segmentation effect with less fluctuation on both healthy and diseased retinal images, and the generalization of the model is strong.

#### 4.6.2 Crossover dataset testing

**Fig 9:**
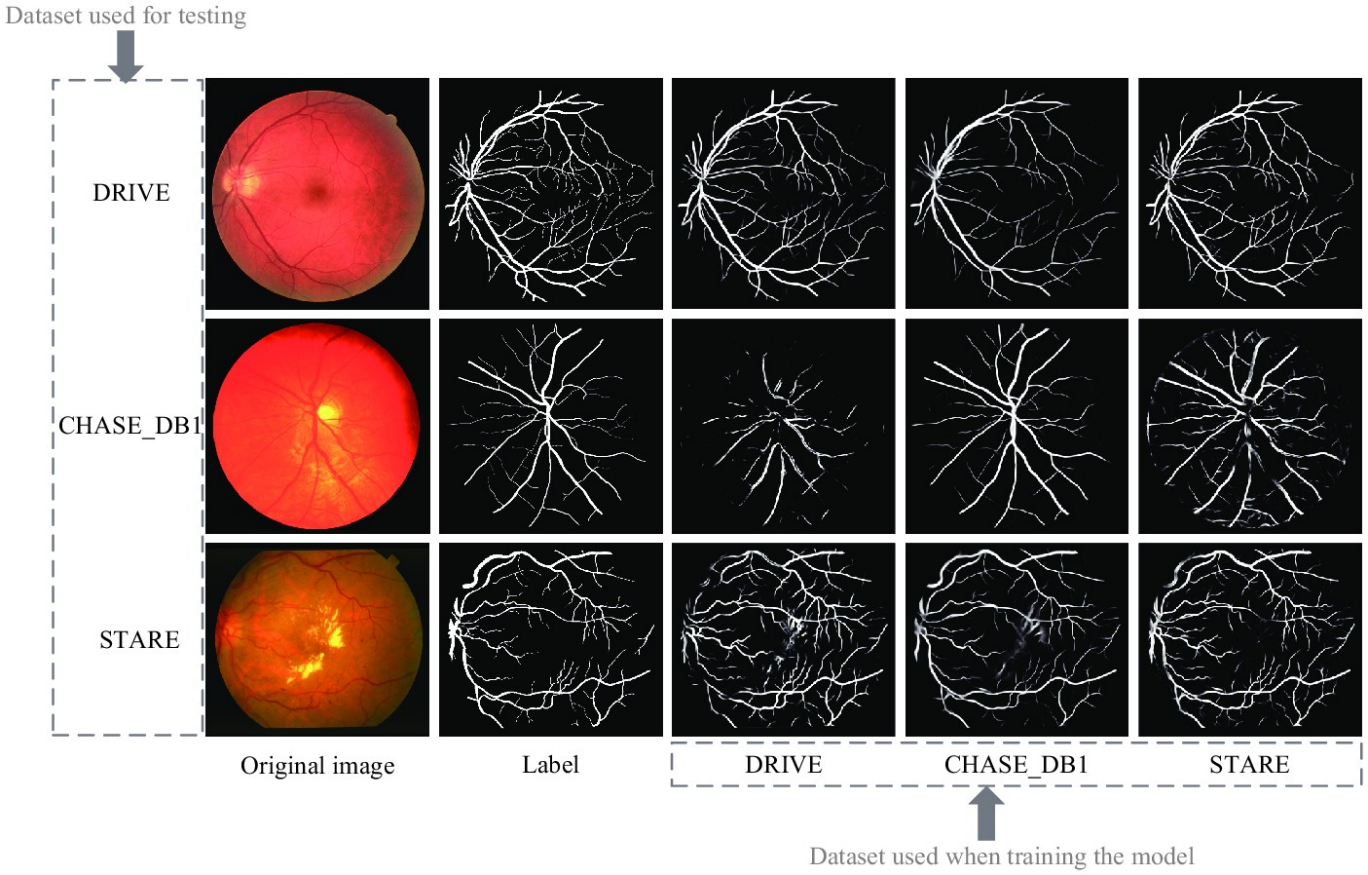
Segmentation result of cross-validate experiment. The first column is the original image; The third to fifth columns are the results of segmenting the three data sets using the model files trained on DRIVE, CHASE DB1 and STARE by MFI-Net.

Retinal images are acquired in different ways, and the three datasets used in the experiments used their own different acquisition methods. Therefore, the retinal images used in clinical practice often have differences in illumination, noise, etc. To verify the generalization ability of MFI-Net on different datasets, we designed a cross-test experiment between multiple datasets. The so-called cross-testing is to train the model on one dataset and then test it on another dataset. Table 7 shows the results of the cross-test, where the horizontal table header is the dataset used for training and the vertical table header indicates the dataset used for testing. To visualize the test results, we visualized the segmentation results as shown in Fig 9.

**Table 7:** Cross test results.

	F1			ACC		
	DRIVE	CHASE_DB1	STARE	DRIVE	CHASE_DB1	STARE
**DRIVE**	0.8318	0.6597	0.7933	0.9705	0.9529	0.9670
**CHASE_DB1**	0.4076	0.8150	0.6052	0.9459	0.9762	0.9553
**STARE**	0.7766	0.7861	0.8483	0.9665	0.9694	0.9766

The F1 and accuracy of the models trained on the DRIVE dataset decreased by 5.52% and 0.4% when tested with the STARE dataset, and by 42.52% and 2.46% when tested with CHASE_DB1. The F1 and accuracy of the model trained on the STARE dataset decreased by 5.30% and 0.96% when tested with the DRIVE dataset, and by 24.11% and 2.13% when tested with CHASE_DB1. The F1 and accuracy of the models trained on the CHASE_DB1 dataset decreased by 15.33% and 2.33% when tested with the DRIVE dataset, and by 2.98% and 0.68% when tested with STARE.

The CHASE_DB1 dataset contains more feature information because the sample images are affected by illumination, and the variability between samples is larger, and STARE can extract a lot of useful feature information because the overall variability between sample images is relatively small, but the lesion areas are significantly different. The DRIVE dataset, on the other hand, can extract fewer general features because of the smaller image size on the one hand and the smaller differences between individual samples on the other. Therefore, the models trained by CHASE_DB1 and STARE are relatively more general, with better generalization ability and lower drop in each metric during cross-testing, while the network models trained by DRIVE have more obvious individualization and higher drop in each metric during cross-testing. The above situation can also be seen in the segmentation results plot in Fig 9.

To evaluate the test results, we compared with the work done by others in this area, but since most of the work was cross-tested only on STARE and DRIVE, only these two datasets were compared, and the results are shown in Table 8, the bold part of the table is the best result. Since MFI-Net focuses on the extraction of coarse-grained feature information at a shallow level, the results obtained by our method trained on the STARE dataset, where the original feature information is more adequate and tested on the DRIVE dataset are very impressive, with a 0.88% improvement in the F1 over the method of Jiang et al. [26]. In contrast, the M3FCN [26] network model proposed by Jiang et al. is a multi-path network model that can extract more fine-grained feature information, and thus achieves good results on the DRIVE dataset, which has relatively little shallow information, with a 1.10% improvement in F1 over our method. Combining all evaluation metrics as a whole, our method still has strong competitive power compared with most other methods. The cross-test results show that our method still has an excellent performance in the face of retinal images acquired by different image acquisition devices.

**Table 8:** Results of cross-testing on DRIVE and STARE dataset.

	DRIVE				STARE			
	F1	Acc	Sen	Spe	F1	Acc	Sen	Spe
DUNet [37]	-	0.9481	0.6505	0.9914	-	0.9445	0.8419	0.9563
Fraz et al. [43]	-	0.9456	0.7242	0.9792	-	0.9495	0.7010	0.9770
Li et al. [44]	-	0.9486	**0.7273**	0.9810	-	0.9545	0.7027	0.9828
Yan et al. [45]	-	0.9444	0.7014	0.9802	-	0.9580	0.7319	**0.9840**
M3FCN [26]	0.7845	0.9665	0.6950	**0.9926**	**0.7876**	0.9647	**0.8604**	0.9733
MFINet(Ours)	**0.7933**	**0.9670**	0.7248	0.9920	0.7766	**0.9665**	0.8342	0.9742

#### 4.6.3 Shortcomings of current work and future research directions

Our work is dedicated to improving the segmentation accuracy of segmentation networks of retinal vessels while discussing the performance of the network model from several aspects. However, we still have the following shortcomings: (1) In order to improve the segmentation accuracy of the network, we use more time and storage as swaps, which makes our network model highly demanding on the hardware. (2) Also the datasets used in our work are limited to three public datasets, DRIVE, CHASE_DB1 and STARE, and we did not experiment on more and larger datasets, so we cannot present more convincing evidence to prove the superior performance of our network model. (3) Although we conducted experiments on different data and cross-tests and the results outperformed many other current methods, the segmentation results of the training model obtained using datasets with less noise and focal regions were not as good as expected on the strongly noisy datasets.

Based on these shortcomings, we have developed directions and priorities for continued research in the future. First, we will continue to optimize our deep learning approach by optimizing the network structure, adding blocks to the network segmentation speed, and optimizing the generalization ability of the network model to make it possible for production and clinical applications. Second, there are often differences in the retinal vascular images used in clinical diagnosis due to different acquisition devices, and using new pre-processing methods to resolve these differences caused by illumination, etc., and building a network model with stronger generalization ability will be of great significance in clinical applications.

## 5 Conclusion

The MFI-Net retinal vessel segmentation network model proposed in this paper enhances the performance of the network model by enhancing the fusion of semantic information. The multi-resolution input module enhances the extraction and transmission of feature information in the shallow layer, which alleviates the problems of blurred boundaries of segmentation results and inaccurate capillary segmentation. The redesigned skip connection part makes the information transfer between the deep and shallow layers of the network more smooth, and richer feature information is fully used in the information fusion process of each layer, which greatly reduces the noise in the segmented images. We tested the MFI-Net network model proposed in this paper on DRIVE, CHASE_DB1 and STARE datasets, and the accuracy reached 97.05%, 97.62% and 97.66%, respectively, and the F1 reached 83.18%, 81.56% and 84.83%, respectively. Experiments were also designed to analyze and demonstrate that MFI-Net has better stability and generalization ability. By analyzing and comparing the segmentation results and discussing the robustness of the model, the MFI-Net network model proposed in this paper has more advantages compared with other methods.

## References

[1] Feng Heyang. Research on Image Segmentation Algorithm of Retinal Vessels[J]. Southwest Jiaotong University, 2017: 1–z.

[2] Soomro TA, Khan TM, Khan M A U, et al. Impact of ICA-based image enhancement technique on retinal blood vessels segmentation[J]. IEEE Access, 2018, 6: 3524–3538. doi: 10.1109/ACCESS.2018.2794463

[3] Staal J, Abràmoff M D, Niemeijer M, et al. Ridge-based vessel segmentation in color images of the retina[J]. IEEE transactions on medical imaging, 2004, 23(4): 501–509. doi: 10.1109/TMI.2004.825627 15084075

[4] Zhang B, Zhang L, Zhang L, et al. Retinal vessel extraction by matched filter with first-order derivative of Gaussian[J]. Computers in biology and medicine, 2010, 40(4): 438–445. doi: 10.1016/j.compbiomed.2010.02.008 20202631

[5] Wang XH, Zhao YQ, Liao M, et al. Automatic segmentation for retinal vessel based on multi-scale 2D Gabor wavelet[J]. Acta Automatica Sinica, 2015, 41(5): 970–980.

[6] Oliveira WS, Teixeira JV, Ren TI, et al. Unsupervised retinal vessel segmentation using combined filters[J]. PloS one, 2016, 11(2): e0149943. doi: 10.1371/journal.pone.0149943 26919587 PMC4769136

[7] Liskowski P, Krawiec K. Segmenting retinal blood vessels with deep neural networks[J]. IEEE transactions on medical imaging, 2016, 35(11): 2369–2380. doi: 10.1109/TMI.2016.2546227 27046869

[8] Aslani S, Sarnel H. A new supervised retinal vessel segmentation method based on robust hybrid features[J]. Biomedical Signal Processing and Control, 2016, 30: 1–12. doi: 10.1016/j.bspc.2016.05.006

[9] Marín D, Aquino A, Gegúndez-Arias M E, et al. A new supervised method for blood vessel segmentation in retinal images by using gray-level and moment invariants-based features[J]. IEEE Transactions on medical imaging, 2010, 30(1): 146–158. 20699207 10.1109/TMI.2010.2064333

[10] Dharmawan DA, Li D, Ng BP, et al. A new hybrid algorithm for retinal vessels segmentation on fundus images[J]. IEEE Access, 2019, 7: 41885–41896. doi: 10.1109/ACCESS.2019.2906344

[11] Ronneberger O, Fischer P, Brox T. U-net: Convolutional networks for biomedical image segmentation[C]//International Conference on Medical image computing and computer-assisted intervention. Springer, Cham, 2015: 234-241.

[12] Alom MZ, Hasan M, Yakopcic C, et al. Recurrent residual convolutional neural network based on u-net (r2u-net) for medical image segmentation[J]. arXiv preprint arXiv:1802.06955, 2018.

[13] Zhang B, Huang S, Hu S. Multi-scale neural networks for retinal blood vessels segmentation[J]. arXiv preprint arXiv:1804.04206, 2018.

[14] Li R, Li M, Li J, et al. Connection sensitive attention U-NET for accurate retinal vessel segmentation[J]. arXiv preprint arXiv:1903.05558, 2019.

[15] Ackley DH, Hinton GE, Sejnowski TJ. A learning algorithm for Boltzmann machines[J]. Cognitive science, 1985, 9(1): 147–169. doi: 10.1207/s15516709cog0901_7

[16] Hinton GE, Salakhutdinov RR. Reducing the dimensionality of data with neural networks[J]. science, 2006, 313(5786): 504–507. doi: 10.1126/science.1127647 16873662

[17] Long J, Shelhamer E, Darrell T. Fully convolutional networks for semantic segmentation[C]//Proceedings of the IEEE conference on computer vision and pattern recognition. 2015: 3431-3440. 10.1109/TPAMI.2016.2572683 27244717

[18] Zhou Z, Siddiquee M M R, Tajbakhsh N, et al. Unet++: Redesigning skip connections to exploit multiscale features in image segmentation[J]. IEEE transactions on medical imaging, 2019, 39(6): 1856–1867. doi: 10.1109/TMI.2019.2959609 31841402 PMC7357299

[19] Oktay O, Schlemper J, Folgoc LL, et al. Attention u-net: Learning where to look for the pancreas[J]. arXiv preprint arXiv:1804.03999, 2018.

[20] Cai Z, Fan Q, Feris R S, et al. A unified multi-scale deep convolutional neural network for fast object detection[C]//European conference on computer vision. Springer, Cham, 2016: 354-370.

[21] Chen LC, Papandreou G, Kokkinos I, et al. Deeplab: Semantic image segmentation with deep convolutional nets, atrous convolution, and fully connected crfs[J]. IEEE transactions on pattern analysis and machine intelligence, 2017, 40(4): 834–848. doi: 10.1109/TPAMI.2017.2699184 28463186

[22] Huang G, Chen D, Li T, et al. Multi-scale dense convolutional networks for efficient prediction[J]. arXiv preprint arXiv:1703.09844, 2017, 2.

[23] Zhao H, Shi J, Qi X, et al. Pyramid scene parsing network[C]//Proceedings of the IEEE conference on computer vision and pattern recognitio

[24] Zhao H, Qi X, Shen X, et al. Icnet for real-time semantic segmentation on high-resolution images[C]//Proceedings of the European Conference on Computer Vision (ECCV). 2018: 405-420.

[25] Liu W, Sun Y, Ji Q. MDAN-UNet: Multi-Scale and Dual Attention Enhanced Nested U-Net Architecture for Segmentation of Optical Coherence Tomography Images[J]. Algorithms, 2020, 13(3): 60. doi: 10.3390/a13030060

[26] Jiang Y, Zhang H, Tan N, et al. Automatic Retinal Blood Vessel Segmentation Based on Fully Convolutional Neural Networks[J]. Symmetry, 2019, 11(9): 1112. doi: 10.3390/sym11091112

[27] He K, Zhang X, Ren S, et al. Deep residual learning for image recognition[C]//Proceedings of the IEEE conference on computer vision and pattern recognition. 2016: 770-778.

[28] Woo S, Park J, Lee J Y, et al. Cbam: Convolutional block attention module[C]//Proceedings of the European conference on computer vision (ECCV). 2018: 3-19.

[29] Owen CG, Rudnicka AR, Mullen R, et al. Measuring retinal vessel tortuosity in 10-year-old children: validation of the computer-assisted image analysis of the retina (CAIAR) program[J]. Investigative ophthalmology & visual science, 2009, 50(5): 2004–2010. doi: 10.1167/iovs.08-3018 19324866

[30] Hoover AD, Kouznetsova V, Goldbaum M. Locating blood vessels in retinal images by piecewise threshold probing of a matched filter response[J]. IEEE Transactions on Medical imaging, 2000, 19(3): 203–210. doi: 10.1109/42.845178 10875704

[31] Kohavi R. A study of cross-validation and bootstrap for accuracy estimation and model selection[C]//Ijcai. 1995, 14(2): 1137–1145.

[32] Ruder S. An overview of gradient descent optimization algorithms[J]. arXiv preprint arXiv:1609.04747, 2016.

[33] Loshchilov I, Hutter F. Decoupled weight decay regularization[J]. arXiv preprint arXiv:1711.05101, 2017.

[34] Mou L, Zhao Y, Chen L, et al. CS-Net: channel and spatial attention network for curvilinear structure segmentation[C]//International Conference on Medical Image Computing and Computer-Assisted Intervention. Springer, Cham, 2019: 721-730.

[35] Wu Y, Xia Y, Song Y, et al. Vessel-Net: retinal vessel segmentation under multi-path supervision[C]//International Conference on Medical Image Computing and ComputerAssisted Intervention. Springer, Cham, 2019: 264-272.

[36] Jiang Y, Tan N, Peng T, et al. Retinal vessels segmentation based on dilated multi-scale convolutional neural network[J]. IEEE Access, 2019, 7: 76342–76352. doi: 10.1109/ACCESS.2019.2922365

[37] Jin Q, Meng Z, Pham TD, et al. DUNet: A deformable network for retinal vessel segmentation[J]. Knowledge-Based Systems, 2019, 178: 149–162. doi: 10.1016/j.knosys.2019.04.025

[38] Laibacher T, Weyde T, Jalali S. M2u-net: Effective and efficient retinal vessel segmentation for real-world applications[C]//Proceedings of the IEEE Conference on Computer Vision and Pattern Recognition Workshops. 2019: 0-0.

[39] Wang K, Zhang X, Huang S, et al. CTF-Net: Retinal Vessel Segmentation via Deep Coarse-To-Fine Supervision Network[C]//2020 IEEE 17th International Symposium on Biomedical Imaging (ISBI). IEEE, 2020: 1237-1241.

[40] Lv Y, Ma H, Li J, et al. Attention Guided U-Net With Atrous Convolution for Accurate Retinal Vessels Segmentation[J]. IEEE Access, 2020, 8: 32826–32839. doi: 10.1109/ACCESS.2020.2974027

[41] Khan TM, Alhussein M, Aurangzeb K, et al. Residual Connection-Based Encoder Decoder Network (RCED-Net) for Retinal Vessel Segmentation[J]. IEEE Access, 2020, 8: 131257–131272. doi: 10.1109/ACCESS.2020.3008899

[42] Jiang Y, Yao H, Wu C, et al. A Multi-Scale Residual Attention Network for Retinal Vessel Segmentation[J]. Symmetry, 2021, 13(1): 24. doi: 10.3390/sym13010024

[43] Fraz MM, Remagnino P, Hoppe A, et al. Blood vessel segmentation methodologies in retinal images–a survey[J]. Computer methods and programs in biomedicine, 2012, 108(1): 407–433. doi: 10.1016/j.cmpb.2012.03.009 22525589

[44] Li Q, Feng B, Xie LP, et al. A cross-modality learning approach for vessel segmentation in retinal images[J]. IEEE transactions on medical imaging, 2015, 35(1): 109–118. doi: 10.1109/TMI.2015.2457891 26208306

[45] Yan Z, Yang X, Cheng KT. A three-stage deep learning model for accurate retinal vessel segmentation[J]. IEEE journal of Biomedical and Health Informatics, 2018, 23(4): 1427–1436. doi: 10.1109/JBHI.2018.2872813 30281503

